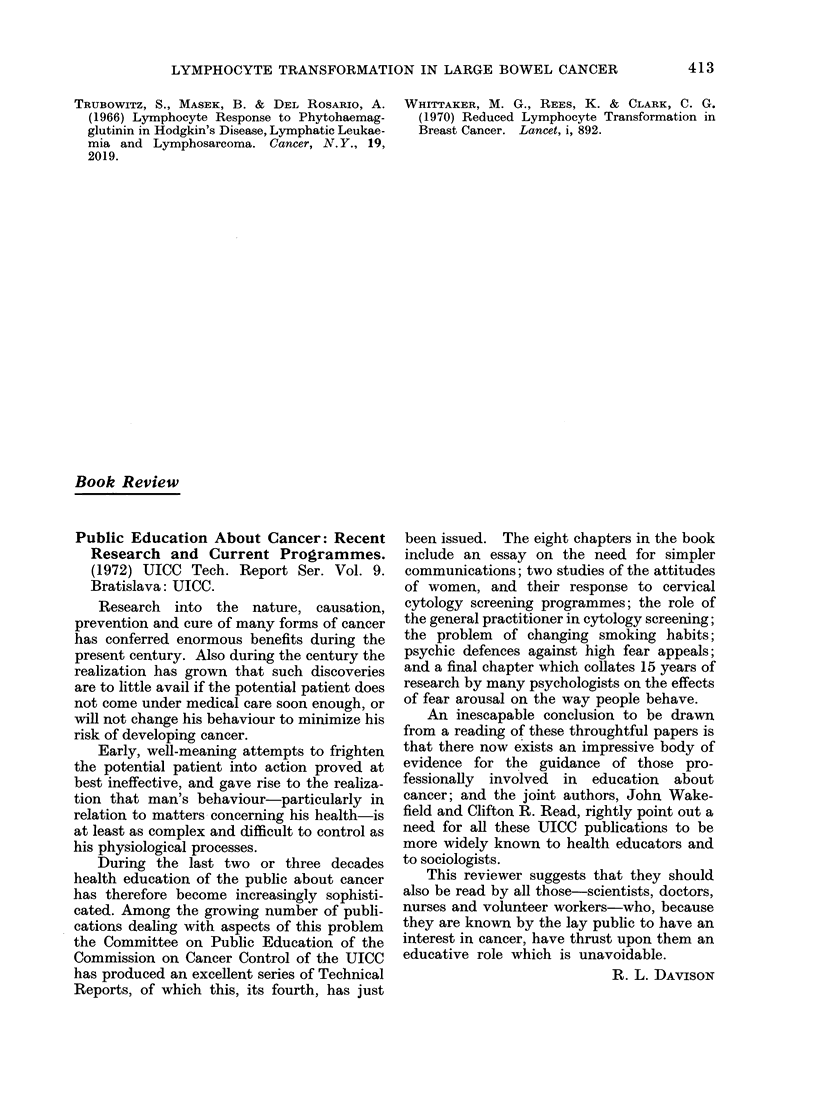# Public Education About Cancer: Recent Research and Current Programmes

**Published:** 1973-05

**Authors:** R. L. Davison


### *Book Review*

**Public Education About Cancer: Recent Research and Current Programmes.** (1972) UICC Tech. Report Ser. Vol. 9. Bratislava: UICC.

Research into the nature, causation, prevention and cure of many forms of cancer has conferred enormous benefits during the present century. Also during the century the realization has grown that such discoveries are to little avail if the potential patient does not come under medical care soon enough, or will not change his behaviour to minimize his risk of developing cancer.

Early, well-meaning attempts to frighten the potential patient into action proved at best ineffective, and gave rise to the realization that man's behaviour—particularly in relation to matters concerning his health—is at least as complex and difficult to control as his physiological processes.

During the last two or three decades health education of the public about cancer has therefore become increasingly sophisticated. Among the growing number of publications dealing with aspects of this problem the Committee on Public Education of the Commission on Cancer Control of the UICC has produced an excellent series of Technical Reports, of which this, its fourth, has just been issued. The eight chapters in the book include an essay on the need for simpler communications; two studies of the attitudes of women, and their response to cervical cytology screening programmes; the role of the general practitioner in cytology screening; the problem of changing smoking habits; psychic defences against high fear appeals; and a final chapter which collates 15 years of research by many psychologists on the effects of fear arousal on the way people behave.

An inescapable conclusion to be drawn from a reading of these throughtful papers is that there now exists an impressive body of evidence for the guidance of those professionally involved in education about cancer; and the joint authors, John Wakefield and Clifton R. Read, rightly point out a need for all these UICC publications to be more widely known to health educators and to sociologists.

This reviewer suggests that they should also be read by all those—scientists, doctors, nurses and volunteer workers—who, because they are known by the lay public to have an interest in cancer, have thrust upon them an educative role which is unavoidable.

R. L. DAVISON